# De Novo Transcriptome Sequencing of the Deep-Sea-Derived Fungus *Dichotomomyces cejpii* and Analysis of Gliotoxin Biosynthesis Genes

**DOI:** 10.3390/ijms19071910

**Published:** 2018-06-29

**Authors:** Wei Ye, Weimin Zhang, Taomei Liu, Zilei Huang, Muzi Zhu, Yuchan Chen, Haohua Li, Saini Li

**Affiliations:** State Key Laboratory of Applied Microbiology Southern China, Guangdong Provincial Key Laboratory of Microbial Culture Collection and Application, Guangdong Open Laboratory of Applied Microbiology, Guangdong Institute of Microbiology, 100 Central Xianlie Road, Yuexiu District, Guangzhou 510070, China; yewei@gdim.cn (W.Y.); ltm840801@163.com (T.L.); yaoyz@gdim.cn (Z.H.); zhumuzi@foxmail.com (M.Z.); chenyc@gdim.cn (Y.C.); Lihh@gdim.cn (H.L.); lisn@gdim.cn (S.L.)

**Keywords:** *Dichotomomyces cejpii*, transcriptome, gliotoxin biosynthesis, glutathione *S*-transferase, aldehyde reductase

## Abstract

Gliotoxin, produced by fungi, is an epipolythiodioxopiperazine (ETP) toxin with bioactivities such as anti-liver fibrosis, antitumor, antifungus, antivirus, antioxidation, and immunoregulation. Recently, cytotoxic gliotoxins were isolated from a deep-sea-derived fungus, *Dichotomomyces cejpii*. However, the biosynthetic pathway for gliotoxins in *D. cejpii* remains unclear. In this study, the transcriptome of *D. cejpii* was sequenced using an Illumina Hiseq 2000. A total of 19,125 unigenes for *D. cejpii* were obtained from 9.73 GB of clean reads. Ten genes related to gliotoxin biosynthesis were annotated. The expression levels of gliotoxin-related genes were detected through quantitative real-time polymerase chain reaction (qRT-PCR). The *GliG* gene, encoding a glutathione *S*-transferase (DC-GST); *GliI*, encoding an aminotransferase (DC-AI); and *GliO*, encoding an aldehyde reductase (DC-AR), were cloned and expressed, purified, and characterized. The results suggested the important roles of DC-GST, DC-AT, and DC-AR in the biosynthesis of gliotoxins. Our study on the genes related to gliotoxin biosynthesis establishes a molecular foundation for the wider application of gliotoxins from *D. cejpii* in the biomedical industry in the future.

## 1. Introduction

Gliotoxin is an epipolythiodioxopiperazine (ETP) toxin with bioactivities such as anti-liver fibrosis, antitumor, antifungus, antivirus, antioxidation, and immunoregulation. It was reported that gliotoxin could inhibit the proliferation of cancer cells and the replication of virus RNAs, and induced the apoptosis of hepatic stellate cells, as well as suppressing the expression of transcription factor NF-κB to reduce the symptom of inflammation [1,2,3]. Gliotoxins have broad application prospects in the biomedical industry and agriculture. A synthetic analog of the natural gliotoxin derivative produced by marine-derived *Aspergillus* sp. CNC-139, named as plinabulin, has entered phase III clinical study for the treatment of nonsmall cell lung cancer [4]. Gliotoxin has also been developed as an antifungal agent to prevent rice BLAST and banded sclerotial blight [5]. It also has been demonstrated that the presence of gliotoxin can promote the survival rate when *A. fumigatus* is exposed to high levels of H_2_O_2_ [6].

Gliotoxins have been isolated from different fungi, including *Aspergillus* sp. [6,7], *Trichoderma virens* [8], *Gliocladium fumbriatum* [9], *Eurotium* sp. [10], *Penicillium* sp. [11], and *D. cejpii* [12]. The biosynthetic pathway of gliotoxins in *A. fumigatus* has been elucidated. The *gli* cluster related to the gliotoxin biosynthesis in *A. fumigatus* was identified [13,14,15], which consists of 13 genes. It has been demonstrated that *GliG* encodes a glutathione *S*-transferase (GST), which conjugates two glutathione (GSH) molecules to form a bis-glutathionylated biosynthetic intermediate, and which is responsible for the sulfurization of gliotoxin, demonstrating that GSTs play an important biosynthetic role in the fungus [13]. *GliI* encodes an aminotransferase, which removes the amino groups in the side chain during the process of gliotoxin biosynthesis. *GliO* encodes an aldehyde reductase in *A. fumigatus*, which is characterized by a broad substrate specificity and a great preference of the reduction reaction [13].

In our previous study, cytotoxic gliotoxins with new structures were isolated from a deep-sea-derived fungus, *Dichotomomyces cejpii* FS110, which showed anticancer activity towards cancer cells including HepG-2, MCF-7, and SF-268 [12]; and the ITS (Internal Transcribed Spacer) sequence of *D. cejpii* showed an identity of 93% with that of *A. fumigatus*. However, the biosynthetic mechanism of gliotoxins in *D. cejpii* remains unclear. It is necessary to investigate the biosynthetic pathway of gliotoxins in *D. cejpii.* In this study, the transcriptome of *D. cejpii* was sequenced using the Illumina sequencing platform. Genes related to gliotoxin biosynthesis in *D. cejpii* including *GliG*, *GliI*, and *GliO* were expressed, purified, and characterized. This is the first report on the characterization of the complete transcriptome of the deep-sea fungus *D. cejpii* and revealing genes related to gliotoxin biosynthesis in *D. cejpii*. Moreover, the elucidation of genes related to gliotoxin biosynthesis will establish a foundation for the metabolic engineering of *D. cejpii* to promote the discovery of new bioactive gliotoxins, thus facilitating the future application of gliotoxins in the biomedical industry.

## 2. Results

### 2.1. Sequencing and De Novo Assembly

In summary, 115,692,236 sequence reads with Q20 value of 97.70% and GC percent of 53.38% were generated and a total of 9,715,411,080 (9.49 GB) nucleotides was obtained, and raw data were deposited in the NCBI (National Center for Biotechnology Information) database with the accession number SRR113435. The short reads were assembled, and 19,301 contigs with a mean length of 1048 bp and 19,125 unigenes with a mean length of 2946 bp were obtained. A total of 7230 unigenes with lengths greater than 3000 bp were found. The length distribution of the *D. cejpii* FS110 transcriptome is shown in Figure S1, and the coding sequences (CDS) of the *D. cejpii* FS110 transcriptome is also shown (Figure S2). In total, 1048 CDS with sequence lengths of more than 3000 nt were found.

### 2.2. Functional Annotation and Classification

**Figure 1:**
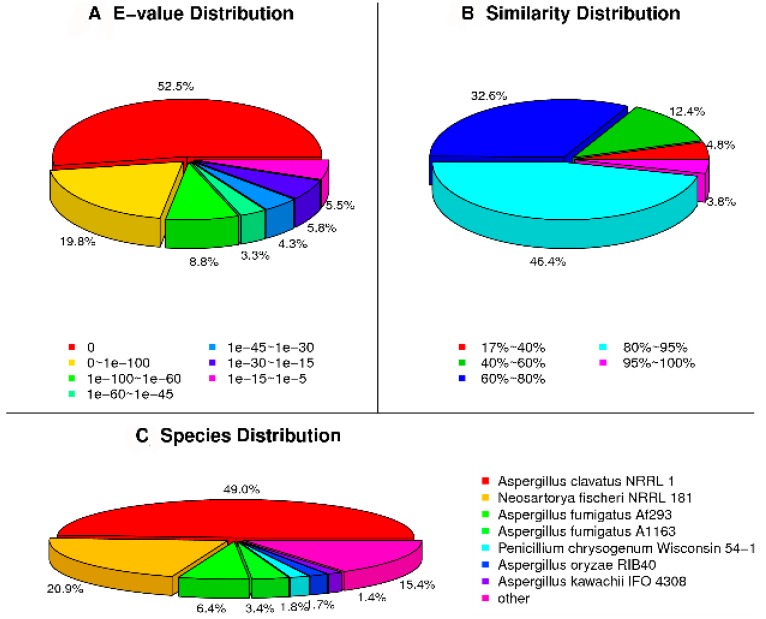
The NR classification of the *D. cejpii* FS110 transcriptome: (**A**) The E-value distribution; (**B**) The similarity distribution; (**C**) The species distribution.

All the unigenes were annotated by public databases, including NR (non-redundant), NT, Swissprot, KEGG (Kyoto Encyclopedia of Genes and Genomes), COG (Cluster of Orthologous Groups of proteins), and GO (Gene Ontology Consortium); 15,196 unigenes were annotated. The unigene number of *D. cejpii* FS110 annotated in different public databases is shown in Table S1; 15,570 unigenes were annotated in the NR database. The NR classification was shown in Figure 1. Overall, 52.5% of the unigenes showed a very high E-value, 17.2% of the unigenes showed similarity of less than 60%, and 32.6% of the unigenes showed similarity of 60–80%. The species distribution results showed that 49.0%, 20.9%, and 6.4% of the unigenes were annotated in *Aspergillus clavatus*, *Neosartoya fischeri*, and *Aspergillus fumigatus*, respectively. The results suggested that there are probably great differences in the *gli* clusters between *A. fumigatus* and *D. cejpii* FS110.

**Figure 2:**
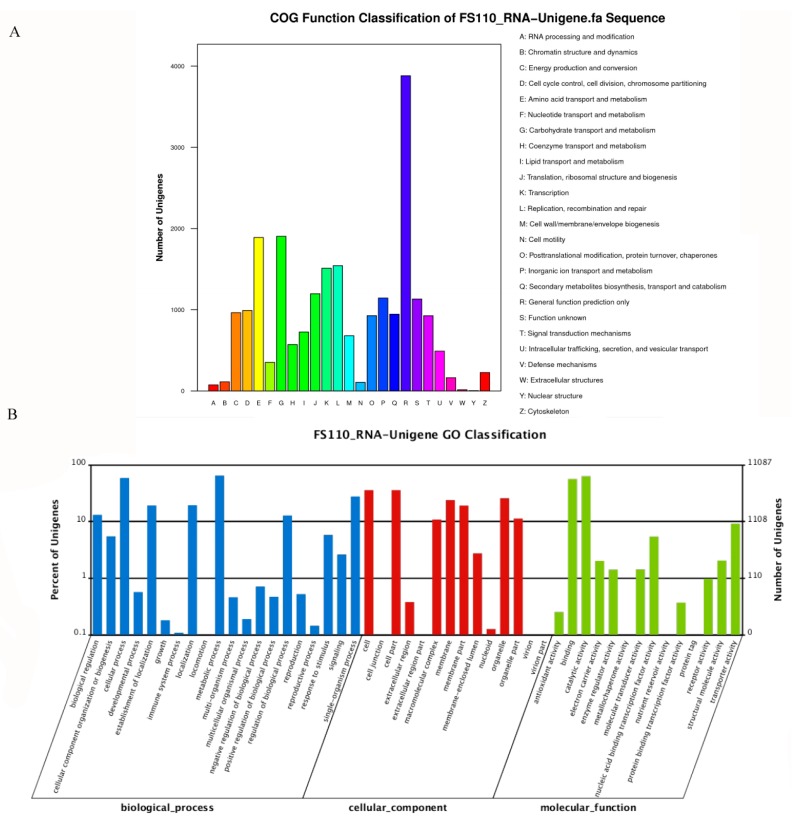
COG (Cluster of Orthologous Groups of proteins) function and GO (Gene Ontology Consortium) function classification of the *D. cejpii* FS110 transcriptome: (**A**) COG functional classification of the *D. cejpii* FS110 transcriptome; (**B**) GO functional classification of the *D. cejpii* FS110 transcriptome.

A total of 15,916 unigenes were annotated and grouped into 25 functional classifications (Figure 2A). The most frequently identified classes were “general function” (3881, 24.4%) and carbohydrate transport and metabolism (1904, 11.9%), followed by amino acid transport and metabolism (1890, 11.8%); transcription (1512, 9.5%); translation (1197, 7.5%); inorganic ion transport and metabolism (1144, 7.2%); function unknown (1132, 7.1%); cell cycle (991, 6.2%); energy production and conversion (964, 6.1%); secondary metabolite biosynthesis, transport, and catabolism (945, 5.9%); and post-translational modification, protein turnover, and chaperones (927, 5.8%). The other 11,087 unigenes were annotated by the GO database and classified into the biological process, cellular component, and molecular function categories (Figure 2B). The high percentage of unigenes involved in the function of catalytic activity indicated the variety of the secondary metabolites produced by *D. cejpii* FS110. To further investigate the biological function of the unigenes, a total of 4569 unigenes were assigned to the metabolic pathways described in the KEGG database, including metabolic pathways, biosynthesis of secondary metabolites, ubiquitin-mediated proteolyis, mitogen-activated protein kinase (MAPK) signaling pathway, and regulation of autophagy RNA transport.

### 2.3. Candidate Genes Involved in Gliotoxin Biosynthesis and Expression Analysis of Five Candidate Genes by qPCR

**Figure 3:**
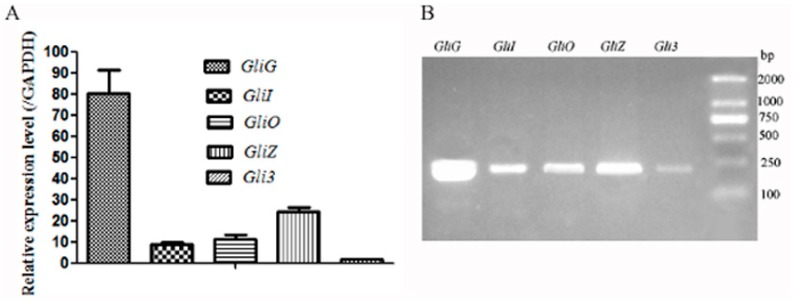
The expression levels of the main unigenes related to the gliotoxin biosynthesis: (**A**) qRT-PCR analysis; (**B**) The identification of qPCR products by electrophoresis.

The expression levels of unigenes, including *GliG* (glutathione *S*-transferase), *GliI* (aminotransferase), *GliO* (aldehyde reductase), *GliZ* (gliotoxin biosynthesis transcription factor), *GliF* (cytochrome P450 oxidoreductase), and *GliP* (nonribosomal peptide synthase), were predicted according to the RPKM (reads per kilobase per million mapped reads) value and validated by qRT-PCR (quantitative real-time polymerase chain reaction) using primers according to the ORFs (Opening reading frames) of the aforementioned genes. The qPCR products for these genes were 200 bp approximately. The *GliG* gene showed the highest expression level, indicating the important role of *GliG* during the biosynthesis of gliotoxins in *D. cejpii*, followed by the genes *GliZ*, *GliO*, *GliI*, and *Gli3*, and *GAPDH* was used as a reference gene. The qPCR products of the *GliG*, *GliO*, *GliI*, *GliZ*, and *Gli3* genes were detected by agarose gel (Figure 3A,B). The sequencing results confirmed that the fragments were desired genes possibly involved in the gliotoxin biosynthesis in *D. cejpii* FS110.

### 2.4. Identification of GliG, GliI, and GliO Genes

**Figure 4:**
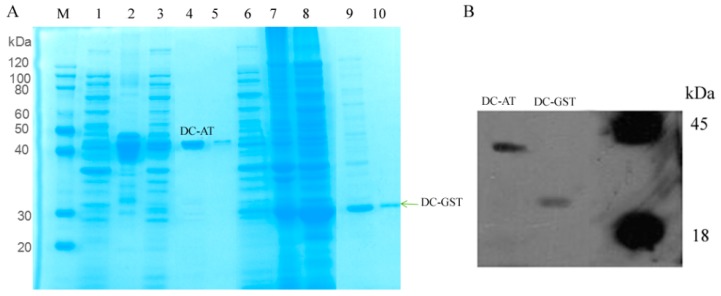
The expression and purification of DC-AT (The aminotransferase encoded by *GliI* from *D. cejpii*) and DC-GST (glutathione *S*-transferase from *D. cejpii*): (**A**) The expression and purification of aminotransferase and glutathione *S*-transferase from *D. cejpii* FS110: M. protein marker; 1. Uninduced sample of AT; 2. Total proteins of induced samples of AT; 3. Supernatant of induced sample of AT; 4. 55 mM imidazole eluate of AT; 5. 100 mM imidazole eluate of AT; 6. Uninduced sample of GST; 7. Total proteins of induced samples of GST; 8. Supernatant of induced sample of GST; 9. 55 mM imidazole eluate of GST; 10. 100 mM imidazole eluate of GST; (**B**) Western blot analysis of DC-AT and DC-GST.

**Figure 5:**
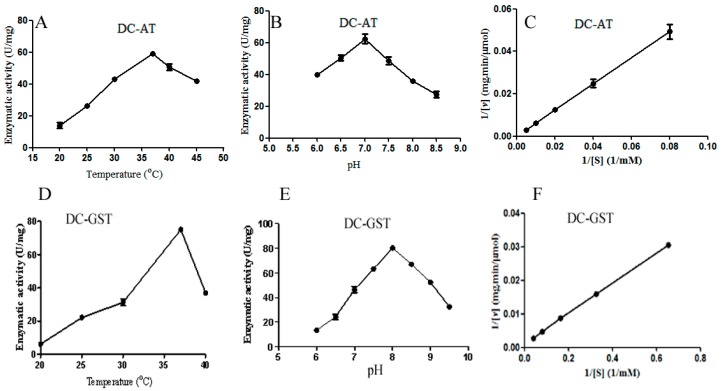
The enzymatic properties of DC-AT and DC-GST: (**A**) The optimal temperature for DC-AT; (**B**) The optimal pH of DC-AT; (**C**) The enzymatic kinetics of DC-AT; (**D**) The optimal temperature for DC-GST; (**E**) The optimal pH of DC-GST; (**F**) The enzymatic kinetics of DC-GST.

Specific primers were designed to amplify the *GliG*, *GliI*, and *GliO* genes with restriction enzyme sites (*Nde*I and *Xho*I), then the amplified fragment was inserted into the vector pET28a, and *GliI* and *GliG* were then expressed in *Escherichia coli* BL21 (DE3), with a molecular weight of 28.0 kDa and 43.5 kDa, respectively. The aminotransferase encoded by *GliI* from *D. cejpii* (DC-AT) and the glutathione *S*-transferase encoded by *GliG* from *D. cejpii* (DC-GST) were purified by Ni-affinity chromatography with a purity of 95.6% and 92.7% in eluate containing 300 mM imidazol, respectively (Figure 4A), and the Western blot analysis result using anti-His monoclonal antibody confirmed the successful expression of the *GliG* gene and *GliI* gene in *E. coli* (Figure 4B). The enzymatic activity of DC-GST was assayed using GSH as substrate. DC-AT showed the highest enzymatic activity of 60.3 ± 1.23 U/mg at the temperature of 37 °C (Fiugre 5A); the optimal pH of DC-AT towards alanine was 7.0, which showed the highest enzymatic activity of 62.26 ± 1.06 U/mg (Figure 5B); and the Km value of DC-AT towards alanine was 523 mM, and the corresponding Vmax was 909 μmol/mg·min (Figure 5C). DC-GST showed the highest enzymatic activity of 75.17 ± 1.67 U/mg at the temperature of 37 °C (Figure 5D), and the optimal pH of DC-GST towards GSH was 8.0 (Figure 5E), which showed the highest enzymatic activity of 80.26 ± 1.12 U/mg. The enzymatic kinetic of DC-GST towards GSH was analyzed, and the Km value of DC-GST towards GSH was 226.5 mM, and the corresponding Vmax was 5 mmol·mL^−1^·min^−1^ (Figure 5F).

**Figure 6:**
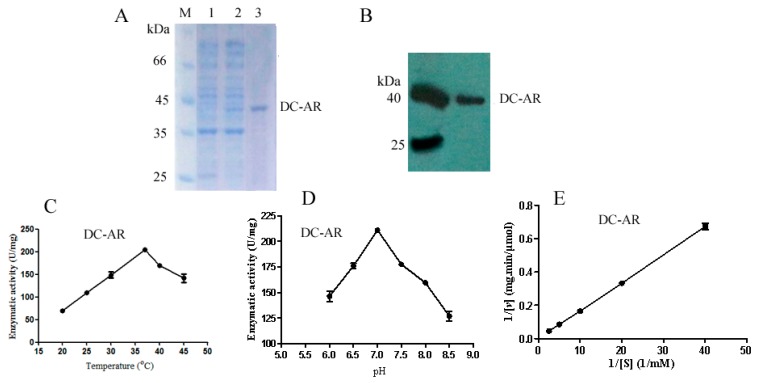
The identification of DC-AR from *D. cejpii*: (**A**) The purification of DC-AR: M, protein marker; 1. Uninduced sample of DC-AR; 2. Induced sample of DC-AR; 3. 100 mM imidazole eluate of DC-AR; (**B**) The Western blot analysis of DC-AR; (**C**) The optimal reaction temperature of DC-AR; (**D**) The optimal reaction pH of DC-AR; (**E**) The enzymatic kinetics of DC-AR.

The aldehyde reductase from *D. cejpii* (DC-AR) encoded by *GliO* was successfully expressed in *E. coli* BL21 (DE3) with a molecular weight of 40.0 kDa (Figure 6A), which was demonstrated by Western blot analysis (Figure 6B). The enzymatic properties of *GliO* were analyzed using p-nitrobenzaldehyde as a substrate. DC-AR showed the highest enzymatic activity of 204.65 ± 3.35 U/mg at the temperature of 37 °C (Figure 6C), the optimal pH for DC-AR towards p-nitrobenzaldehyde was 7.0 (Figure 6D), and the corresponding enzymatic activity was 210.94 ± 1.41 U/mg. The enzymatic kinetics of DC-AR was also investigated. The Km value of DC-AR towards p-nitrobenzaldehyde was 6.4 mM, and the corresponding Vmax was 400 μmol·mg^−1^·min^−1^ (Figure 6E).

### 2.5. Development and Characterization of cDNA-Derived SSR Markers

Different unigenes in *D. cejpii* were identified by MISA analysis, and 19,125 unigenes containing SSRs (simple sequence repeats) were identified (Figure S3), among which 385 sequences contained more than one SSR. Moreover, 97 SSRs were present in the compound formation. The distribution and frequency of the mono-, di-, tri-, tetra-, penta-, and hexanucleotide repeats were analyzed. The most abundant repeat motifs were trinucleotides (990, 33.4%), dinucleotides (743, 25.1%), and mononucleotides (564, 19.0%), followed by hexanucleotides (368, 12.4%) and pentanucleotides (153, 5.2%). The most frequent was AG/CT (563, 19.0%), followed by AAG/CTT (366, 12.4%), ATC/ATG (142, 5.3%), AT/AT (128, 4.3%), AGG/CCT (106, 3.9%), AGC/CTG (97, 3.6%), ACG/CGT (72, 2.8%), AAC/GTT (67, 2.5%), AC/GT (45, 1.7%), ACT/AGT (40, 1.5%), and AAT/ATT (28, 1.0%) (Figure S1).

### 2.6. The Single Nucleotide Polymorphism (SNP) Analysis

The single nucleotide polymorphism (SNP) analysis of the *D. cejpii* FS110 transcriptome was performed. In total, 1956 SNPs were detected in *D. cejpii* FS110 transcriptome data; the most frequent were transition type SNPs (1672, 85.5%), including A–G type (820, 41.9%) and C–T type (852, 43.6%), followed by transversion type (284, 14.5%), including A–C type (92, 4.7%), A–T type (64, 3.3%), C–G type (53, 2.7%), and G–T type (75, 3.8%). The SNP distribution results revealed that the abundant SNPs might result in the phenotypic modulation of *D. cejpii* FS110.

## 3. Discussion

*D. cejpii* is a deep-sea-derived fungus that could produce gliotoxins, which are a kind of DKP (diketopiperazine) with a variety of bioactivities such as anticancer, antifungal, antivirus, antioxidation, and immunoregulation activities [13,16]. The functional mechanisms of gliotoxins include the inhibition of viral reverse transcriptase, binding to alcohol dehydrogenases, the inhibition of NF-κB, the modulation of calcium, and the toxic effects on erythrocytes. Gliotoxin was also reported as a pathogenic factor for *A. fumigatus* [16]. The exposure of mammalian cells to gliotoxin elevates the levels of ROS (reactive oxygen species). Gliotoxin isolated from marine fungus *Aspergillus* sp. was also reported to induce the apoptosis of human cervical cancer and chondrosarcoma cells via the mitochondrial pathway [7]. Hence, gliotoxins have been used as a immunosuppressive agent, tumor suppressor, pesticide, and fungicide [4,16,17,18]. The elevated GSH could promote the anticancer activity of gliotoxins. The transcriptomic data was employed to identify the important gliotoxin biosynthesis-related gene *GliT* [19], which is required for self-protection against the toxins, and the transcriptome data was also utilized to identify the gene related to the biosynthesis of trichothecene toxins in *Myrothecium roridum* [20], suggesting that transcriptome sequencing is an important tool for the investigation of the biosynthetic pathway of gliotoxins in *D. cejpii*. Eleven gliotoxins, including three new gliotoxins, were isolated from *D. cejpii*, implying the unique gliotoxin biosynthesis pathway in *D. cejpii* [12]. Thus, the transcriptome analysis of *D. cejpii* was performed using an Illumina Hiseq 2000^TM^, the genes related to the gliotoxin biosynthesis were annotated and cloned, and the key enzymes for the gliotoxin biosynthesis were identified to elucidate the biosynthetic pathway of gliotoxin in *D. cejpii*, thus laying a foundation for the excavation of new gliotoxins and their derivatives and promoting their application in the biomedical industry in the future.

Using the cDNA of *D. cejpii* as a template, seven kinds of gliotoxin biosynthesis-related genes were obtained, including *GliF*, *GliG*, *GliI*, *GliM*, *GliO*, *GliZ*, and *GliT. GliG*, *GliI*, and *GliO* in *D. cejpii* showed low identities of 31.6%, 28.9%, and 38.7% with those genes in *A. fumigatus*, respectively, indicating the novelty of these genes from *D. cejpii.* The *GliG* gene was annotated as a glutathione *S*-transferase, and GSH was employed as a substrate to evaluate the enzymatic activity of DC-GST. GST from tomato was expressed in *E. coli* with an enzymatic activity of 0.625 U/mg [21,22]; BI-GST (Bax-inhibitor-GST) and other five kinds of tau-class LeGSTs (GST which readily formed heterodimers) expressed in *E. coli* BL21 (DE3) showed enzymatic activities ranging from 0.06 ± 0.01 to 9.22 ± 0.91 U/mg towards CDNB (1-chloro-2,4-dinitrobenzene) [23], which were also much lower than DC-GST, with an enzymatic activity of 80.26 ± 1.12 U/mg. The results indicated that the DC-GST from *D. cejpii* possesses much higher enzymatic activity than that of plant GSTs, perhaps due to the deep-sea environment in which *D. cejpii* lives. It was also reported that the overexpression of a rice tau-class glutathione-*S*-transferase gene in *Arabidopsis* sp. could improve the tolerance to salinity and oxidative stress of *Arabidopsis*, implying that GST is very important for the resistance to adversity in plant and fungi. Furthermore, DC-GST showed the highest reaction velocity of 5 mM·mg^−1^·min^−1^ towards GSH, which is higher than those of other kinds of GSTs [23,24]. It has been demonstrated that GST could remove the excess ROS when *A. fumigatus* is exposed to high concentrations of H_2_O_2_ or when *A. fumigatus* is living in adversity, thus preventing the cells from the harm of ROS [6], indicating the important role of DC-GST for the survival of the deep-sea-derived fungus *D. cejpii* in the deep-sea environment. The enzymatic activity of DC-AT towards alanine indicated the role of DC-AT in the transfer of alanine during the gliotoxin biosynthesis. The DC-AR could catalyze the reduction of p-nitrobenzaldehyde, suggesting the function of DC-AR as an aldehyde reductase in the biosynthesis of gliotoxins and their derivatives.

**Figure 7:**
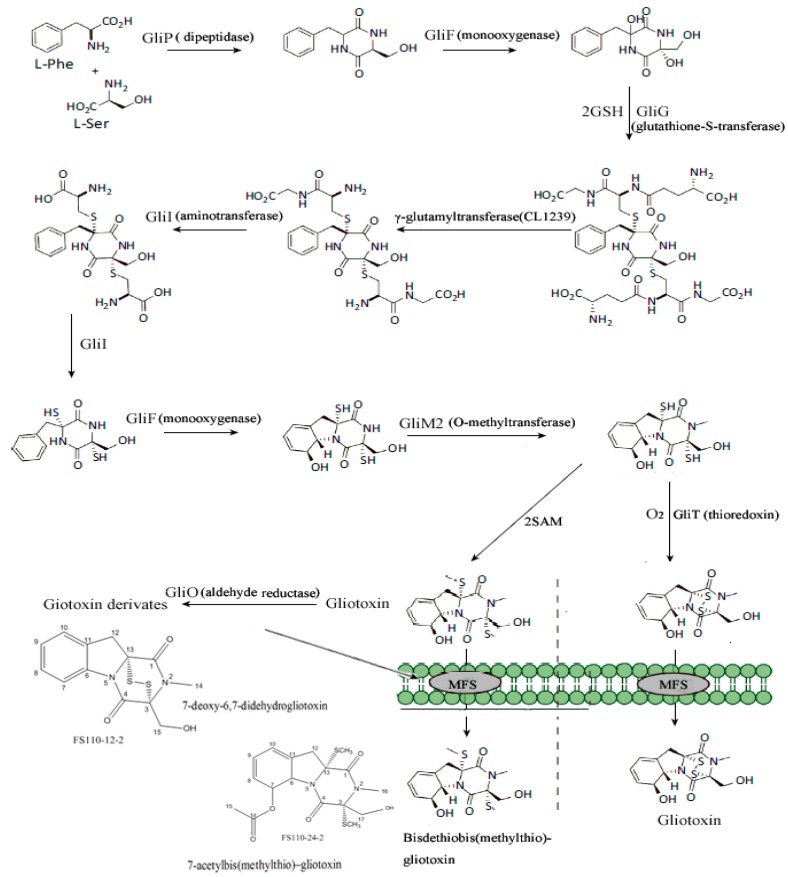
Proposed biosynthesis pathway of gliotoxins in *D. cejpii* FS110 [13]. SAM refers to *S*-adenosyl methionine; MFS refers to major facilitator superfamily.

The gliotoxin biosynthetic pathway in *A. fumigatus* has been elucidated [13,14,15], and it has been demonstrated that *GliG*, *GliI*, and *GliO* play vital roles in the biosynthesis of gliotoxins in *A. fumigatus* [13]. *GliG* is annotated as a glutathione *S*-transferase (GST), which conjugates two glutathione (GSH) molecules to a biosynthetic intermediate to form a bis-glutathionylated biosynthetic intermediate. *GliI* encodes an aminotransferase, which is responsible for the removal of the amino acid in the side chain of intermediates during the biosynthesis of gliotoxin. *GliO* is annotated as an aldehyde reductase related to the gliotoxin formation, which was not involved in the formation of the gliotoxin backbone. In our previous study, different new gliotoxins were obtained, indicating the possible specific biosynthetic pathway of gliotoxins in *D. cejpii*, and that *GliO* was probably involved in the formation of new gliotoxins and their derivatives. The possible biosynthetic pathway of gliotoxins and their derivatives in *D. cejpii* was proposed (Figure 7); *GliP*, *GliF*, *GliG*, *GliI*, *GliM2*, *GliT*, *GliO*, and *MFS* were proposed to be also involved in the biosynthesis of gliotoxins and their derivatives.

This is the first report on characterization of the gliotoxin biosynthesis-related genes in the deep-sea fungus *D. cejpii* FS110, which would lay a foundation for the elucidation of gliotoxin biosynthesis in *D. cejpii* FS110, thereby promoting the wider application of gliotoxins in the biomedical industry. Furthermore, the results would also provide clues for the investigation on the biosynthesis of bioactive compounds isolated from deep-sea fungi.

## 4. Materials and Methods

### 4.1. cDNA Library Construction and RNA Sequencing

The strain *D. cejpii* FS110 (Accession No. KF706672) was isolated from a deep-sea sedimental sample in the South China Sea (19°0.368′ N, 117°58.233′ E; depth 3941 m). *D. cejpii* FS110 was inoculated on a PDA (Potato Dextrose Agar) medium and incubated at 30 °C for 3, 5, 7, and 9 days. The total RNAs of *D. cejpii* FS110 at different growth stages were extracted using an RNA extracting kit (Umagen, Guangzhou, China). The quantity of RNA was checked on agarose gel and the Nanodrop-2000 spectrophotometer (GE, Fairfield, CT, USA). HPLC (high performance liquid chromatography) using an Agilent 2100 (Agilent, Santa Clara, CA, USA) was used to detect the RIN (RNA Integrity Number) value of the total RNA. High-quality RNAs (RIN ≥ 6.0) were selected for high-throughput sequencing. Then, the same amounts of RNAs were mixed, and total RNAs in an amount of 5.0 µg were resuspended in RNase-free water and stored at −80 °C before use. The extracted RNA samples were used for the cDNA synthesis. Poly(A) mRNA was isolated and broken into short fragments (200 nt) by adding a fragmentation buffer. First-strand cDNA was generated using a reverse transcription kit (Abm, Vancouver, BC, Canada). The cDNA fragments were purified using a PCR extraction kit (Tiangen, Guangzhou, China). These purified fragments were ligated to sequencing adapters. Following the agarose gel electrophoresis and extraction of cDNA from gels, the cDNA fragments (200 ± 25 bp) were purified and enriched by PCR to construct the final cDNA library. The cDNA library was sequenced on the Illumina sequencing platform (Illumina HiSeq™ 2000, illumina, San Diego, CA, USA) using the PE (paired-end) technology in a single run. The original image processes of sequencing, base-calling, and quality value calculation were performed by the Illumina GA Pipeline (version 1.6, illumina, San Diego, CA, USA), in which 100-bp paired-end reads were obtained [25,26].

### 4.2. Data Processing, Assembly, and Annotation

To assemble the entire transcriptomes of the different samples better, a PE 100 sequencing strategy was used. All sequences were examined to ensure their accuracy. A Perl program was written to select clean reads by removing low-quality sequences (more than 50% bases with quality lower than 20 in one sequence and Q30 value less than 80% were identified), reads with more than 5% N bases (bases unknown), and reads containing adaptor sequences. Subsequently, the clean reads were assembled using the Trinity software (version 1.4, Campton, NH, USA) to construct unique consensus sequences. Adaptor and low-quality sequences were trimmed. Short sequences (<50 bp) were removed using a custom Perl program (version 5.14, Philadelphia, PA, USA). The resulting high-quality sequences were deposited in the National Center for Biotechnology Information (NCBI) database and de novo assembled into contigs and transcripts. To reduce data redundancy, transcripts with a minimum length of 200 bp were assembled and clustered using the CLC NGS Cell software (version 1.3, Illumina, San Diego, CA, USA) under default parameters. The longest sequences in each cluster were reserved and designated as unigenes. Searches were performed using local BLASTX programs (NCBI, NIH, MD, USA) against sequences in the NR database and the SWISS-PROT database (the E-value cutoff was 1.00 × 10^−5^) [27]. Unigenes were identified according to top hits against known sequences. The resulting unigenes were assigned to GO and COG terms.

### 4.3. KEGG Pathway Analysis and Predicted CDS

Pathway assignments were made according to KEGG mapping. Enzyme commission numbers were assigned to unique sequences that had the best BLASTX scores with cutoff E-values of 1.00 × 10^−5^, as determined from our KEGG database search. The sequences were mapped to the KEGG biochemical pathways according to the Enzyme Commission (EC) distribution within the pathway database.

Then, CDS (coding domain sequences) were extracted based on BLAST results and then translated into peptide sequences. Moreover, BLAST result information was also used to train ESTScan [28]. The CDS of unigenes that had no hit in BLAST were predicted by ESTScan and then translated into peptide sequences.

### 4.4. Quantitative Real-Time Polymerase Chain Reaction Analysis

qRT-PCR (quantitative real-time polymerase chain reaction) was performed to verify the expression levels of unigenes related to the biosynthesis of gliotoxin, predicted according to the RPKM value, using a real-time PCR instrument (Eppendorf, Westbury, NY, USA) with 2SYBR Green mix (Fermentas, Harrington, DE, USA) according to the manufacturer’s instructions. *D. cejpii* FS110 was cultured on PDA medium under 30 °C for seven days, and RNA was extracted. First-strand cDNA was synthesized from 1 μg of total RNA with reverse transcription kit (Abm, Vancouver, BC, Canada), and the resulting products were used as templates for qRT-PCR. The specific primers used for qRT-PCR are listed in Table S2. The qRT-PCR thermal cycling condition for all reactions was 95 °C for 1 min 50 s, followed by 40 cycles at 95 °C for 10 s and 55 °C for 33 s. All reactions were conducted in biological triplicates, and the results were expressed as relative expression levels to the *GAPDH* gene. The *C*_t_ (cycles threshold) values obtained were used as the original data to calculate the relative expression level of different genes to *GAPDH* gene by the 2^−ΔΔ*C*t^ method [29,30,31]. Each sample was analyzed in biological triplicate.

### 4.5. Identification of GliG, GliI, and GliO Genes

The *GliG*, *GliI*, and *GliO* genes were amplified with restriction enzyme *Nde*I and *Xho*I recognition sites using primers listed in Table S3, then inserted into the expression vector pET28a and transformed with *E. coli*, and the recombinant vectors pET28a-*GliG*, pET28a-*GliI*, and pET28a-*GliO* were expressed in *E. coli* BL21 (DE3) after being induced using 1 mM of IPTG for 3.5 h. A supernatant of 200 mL fermentation liquids containing recombinant plasmids after sonication was loaded onto the Ni-affinity chromatography column. The target proteins were eluted with 20 mM Tris–HCl (pH 8.0) containing different concentrations of imidazole. Different protein samples were identified by sodium dodecyl sulfate polyacrylamide gel electrophoresis and transferred to the NC (nitrocellulose) membrane. After being blocked by 5% nonfat milk, the membrane was incubated with mouse anti-His monoclonal antibody (Earthox, Millbrae, CA, USA) and goat anti-mouse IgG antibody (Promega, Madison, WI, USA), and target bands were finally visualized using an ECL (Enhanced Chemiluminescent) kit (Fermentas, Harrington, DE, USA) following the manufacturer’s instructions.

### 4.6. Enzymatic Activity Assay of Enzymes Encoded by GliG, GliI, and GliO

Briefly, l-glutathione (GSH) substrate was mixed with CDNB with a volume ratio of 10:1; 10 µL CDNB was added into 100 µL GSH; the mixture was preincubated with 10 µL DC-GST with a concentration of 0.1 mg/mL at 25 °C for 5 min; and the mixture of GSH, CDNB, and DC-GST was incubated at 25 °C and the absorbance at 340 nm was monitored; one unit was defined as the desired amount of DC-GST for catalyzing the combination of 1 µmol CDNB and GSH in one minute. The optimal reaction temperature and reaction pH for DC-GST were investigated at the reaction temperatures of 20, 25, 30, 37, and 40 °C and reaction pH values of 6.0, 6.5, 7.0, 7.5, 8.0, 8.5, 9.0, 9.5, and 10.0. Substrate thioanisole with different concentrations of 24.4, 12.2, 6.1, 3.05, and 1.525 mM were used to investigate the enzymatic kinetics of DC-GST [32].

l-alanine and α-oxoglutarate were employed as substrates to assay the enzymatic activity of DC-AT. Briefly, 4 μL DC-AT with the concentration of 0.15 mg/mL was added to 15 μL of substrates containing 50 mM α-oxoglutarate and 200 mM l-alanine, and without the substrate addition into the blank group; then the mixtures and blank group were incubated at 37 °C for 30 min. The substrates were added to the blank group after the incubation, 15 μL dinitrophenylhydrazine was added to the mixture and incubated at 37 °C for 20 min, 0.1 M Tris-HCl buffer (pH 8.0) was added, and the absorbance at 505 nm was detected. One unit was defined as the desired amount of DC-AT for the production of 1 µmol of pyruvic acid in one minute. The optimal reaction temperature and reaction pH for DC-GST were investigated at the reaction temperatures of 20, 25, 30, 37 °C, and 40 °C and reaction pH values of 6.0, 6.5, 7.0, 7.5, 8.0, 8.5, 9.0, 9.5, and 10.0. Substrate l-alanine with different concentrations of 200, 100, 50, 25 mM, and 12.5 mM were used to investigate the enzymatic kinetics of DC-AT.

*p*-Nitrobenzaldehyde and ethyl 4-chloroacetoacetate were used as substrates to investigate the enzymatic properties of DC-AR. Briefly, 20 µL 4-COBE and 20 µL p-nitrobenzaldehyde with a concentration of 0.2 mM, 5 µL NAPDH (β-Nicotinamide adenine dinucleotide 2′-phosphate reduced) with a concentration of 0.2 mM, and 5 µL DC-AR with a concentration of 0.1 mg/mL were added to the substrates, and the above mixtures were incubated at 37 °C and the absorbance at 340 nm was monitored; one unit was defined the desired amount of DC-AR for the consumption of 1 µmol of NAPDH in one minute. The optimal reaction temperature and reaction pH for DC-AR were investigated at the reaction temperatures of 20, 25, 30, 37, 40 °C, and 45 °C and the reaction pH values of 6.5, 7.0, 7.5, 8.0, and 8.5. Substrate thioanisole with different concentrations of 0.4, 0.2, 0.1, 0.05 mM, and 0.025 mM were used to investigate the enzymatic kinetics of DC-AR.

### 4.7. Identification of SSRs

The microsatellite identification tool (MISA, available online: http://pgrc.inpk-gatersleben.de/misa/) was used to identify SSRs [33]. The parameters were adjusted to identify perfect di-, tri-, tetra-, penta-, and hexanucleotide motifs with a minimum of 6, 5, 5, 4, and 4 repetitions, respectively. Primer pairs were designed using the Primer 3 software and selected according to the following criteria: (1) primers with SSRs were eliminated; (2) primers aligned with unigene sequences were allowed three mismatches at the 5′ site and one mismatch at the 3′ site; (3) primers that aligned to more than one unigene were eliminated; and (4) SSRs were identified using the SSR_finder (Available online: http://www.fresnostate.edu/ssrfinder/). We kept the products whose results from SSR_finder and MISA were the same.

### 4.8. Identification of SNPs

SNPs (single-nucleotide polymorphism) were called using a short-read alignment algorithm, which aligned nonassembled 50 bp Illumina reads from ‘67/3’ against the ‘305E40’ assembly, by analogy with the MAQ (a pipeline such as MAQ which aligns read data to a reference sequence) style sequence pileup at a minimum coverage of 6×; to call indels, an SSAHA (Sequence Search Hashing Algorithm)-based alignment strategy [34] was applied. Each allele was observed at least three times.

Each SNP was assigned a designability score via a dedicated “assay design tool” (Available online: http://www.illumina.com), which identified SNP loci free of other polymorphisms 60 bp either upstream or downstream. A quality score based on the probability of good performance using the Illumina Golden Gate assay was assigned to each SNP, where a score >0.6 indicated a high probability of success.

## 5. Conclusions

In conclusion, the transcriptome of *D. cejpii* FS110 was firstly sequenced by an Illumina Hiseq 2000 system. The expression levels of genes related to the gliotoxins biosynthesis were analyzed, and *GliG*, *GliI*, and *GliO* were cloned and expressed. The results demonstrated that the *GliG* gene plays an important role in the biosynthesis of gliotoxins in *D. cejpii* FS110. The low identities of *GliG*, *GliI*, and *GliO* in *D. cejpii* FS110 compared with those in *A. fumigatus* suggested the novelty of these genes. The enzymatic properties investigation results demonstrated the important role of *GliG* and *GliI* in the biosynthesis of the gliotoxin backbone and the function of *GliO* in the modification of gliotoxins. The biosynthetic pathway of gliotoxins and their derivatives in *D. cejpii* FS110 were proposed in our study. The SSR and SNP analysis of the *D. cejpii* FS110 transcriptome revealed the gene polymorphism in *D. cejpii* FS110. The above results establish a foundation for the elucidation of the gliotoxin biosynthesis and the excavation of more gliotoxins and their derivatives, thus promoting the wider application of gliotoxins in the biomedical industry in the future.

## Supplementary Materials

Supplementary materials can be found at http://www.mdpi.com/1422-0067/19/7/1910/s1.

## Abbreviations

CDS	Coding domain sequences
COG	Cluster of orthologous groups of proteins
DC-GST	Glutathione-*S*-transferase from *D. cejpii*
DC-AT	Aminotransferase from *D. cejpii*
DC	Aldehyde reductase from *D. cejpii*
ETP	Epipolythiodioxopiperazine
GO	Gene Ontology Consortium
HPLC	High performance liquid chromatography
KEGG	Kyoto Encyclopedia of Genes and Genomes
MAPK	Mitogen-activated protein kinase
qRT-PCR	Quantitative real-time polymerase chain reaction
SSR	Simple sequence repeat
SNP	Single nucleotide polymorphism
