# Supplementary material for: De Novo Transcriptome Sequencing of the Deep-Sea-Derived Fungus Dichotomomyces cejpii and Analysis of Gliotoxin Biosynthesis Genes
Source: Int J Mol Sci. 2018 Jun 29;19(7):1910. doi: 10.3390/ijms19071910 (PMC6073683; doi:10.3390/ijms19071910)
Supplement: Supplementary file 1 [file ijms-19-01910-s001.zip › Table S1 sequences of FS110 annotated in different database.docx]

| Sequence File | NR | NT | Swiss-Prot | KEGG | COG | GO | ALL |
| --- | --- | --- | --- | --- | --- | --- | --- |
| FS110 unigenes | 15,570 | 14,400 | 11,744 | 11,757 | 9,536 | 11,087 | 15,916 |

Table S1 The sequences of FS110 transcriptome annotated in different database
