# Supplementary material for: De Novo Transcriptome Sequencing of the Deep-Sea-Derived Fungus Dichotomomyces cejpii and Analysis of Gliotoxin Biosynthesis Genes
Source: Int J Mol Sci. 2018 Jun 29;19(7):1910. doi: 10.3390/ijms19071910 (PMC6073683; doi:10.3390/ijms19071910)
Supplement: Supplementary file 1 [file ijms-19-01910-s001.zip › Table S2 Primers used for the qRT-PCR of unigenes related to gliotoxin biosynthesis.docx]

Table S2 Primers used for the qRT-PCR of genes related to the gliotoxin biosynthesis in *D.cejpii* FS110

| Genes | Sequences(5’-3’) |
| --- | --- |
| *GliG* F | CGGTCGTAACCTACACGAGAG |
| *GliG* R | GTCAGCCAGGGCCAAGTATTG |
| *GliI* F | CCCTCATCCCGGCTTATATC |
| *GliI* R | GAATCACAGAGAGAGCGTAG |
| *GliO* F | GGGTTATCCAATTTCCGCGC |
| *GliO* R | TTGGTCAGAAACCCACCCCG |
| *GliZ* F | ACGACTCCTCCTCTGCTTCTC |
| *GliZ* R | GTGCCTCTAATGCGTCAACTG |
| *Gli3* F | ATGCGACCGCAGTTTCACCCG |
| *Gli3* R | TTCGTCCTTGGGCTGAGAGAG |
