# Supplementary material for: De Novo Transcriptome Sequencing of the Deep-Sea-Derived Fungus Dichotomomyces cejpii and Analysis of Gliotoxin Biosynthesis Genes
Source: Int J Mol Sci. 2018 Jun 29;19(7):1910. doi: 10.3390/ijms19071910 (PMC6073683; doi:10.3390/ijms19071910)
Supplement: Supplementary file 1 [file ijms-19-01910-s001.zip › Table S3 Primers used for the expression of genes.docx]

Table S3 Primers used for the expression of genes related to the gliotoxin biosynthesis in *D.cejpii* FS110

| Genes | Sequences(5’-3’) |
| --- | --- |
| *GliG* F | GGAATTCCATATGACCGAACGACCTTCTGATC |
| *GliG* R | CCGCTCGAGCAATAGTCCATACTCCTTC |
| *GliI* F | GGAATTCCATATGCCTCACGCAGAAACACTC |
| *GliI* R | CCGCTCGAGCCACCTCTTATCCACCCCC |
| *GliO* F | GGAATTCCATATGGCCAATTCTCGACCCAACATC |
| *GliO* R | CCGCTCGAGGTTGAAGTTATCCAGGATTGCG |
