# Supplementary figures and images for: De Novo Transcriptome Sequencing of the Deep-Sea-Derived Fungus Dichotomomyces cejpii and Analysis of Gliotoxin Biosynthesis Genes
Source: Int J Mol Sci. 2018 Jun 29;19(7):1910. doi: 10.3390/ijms19071910 (PMC6073683; doi:10.3390/ijms19071910)

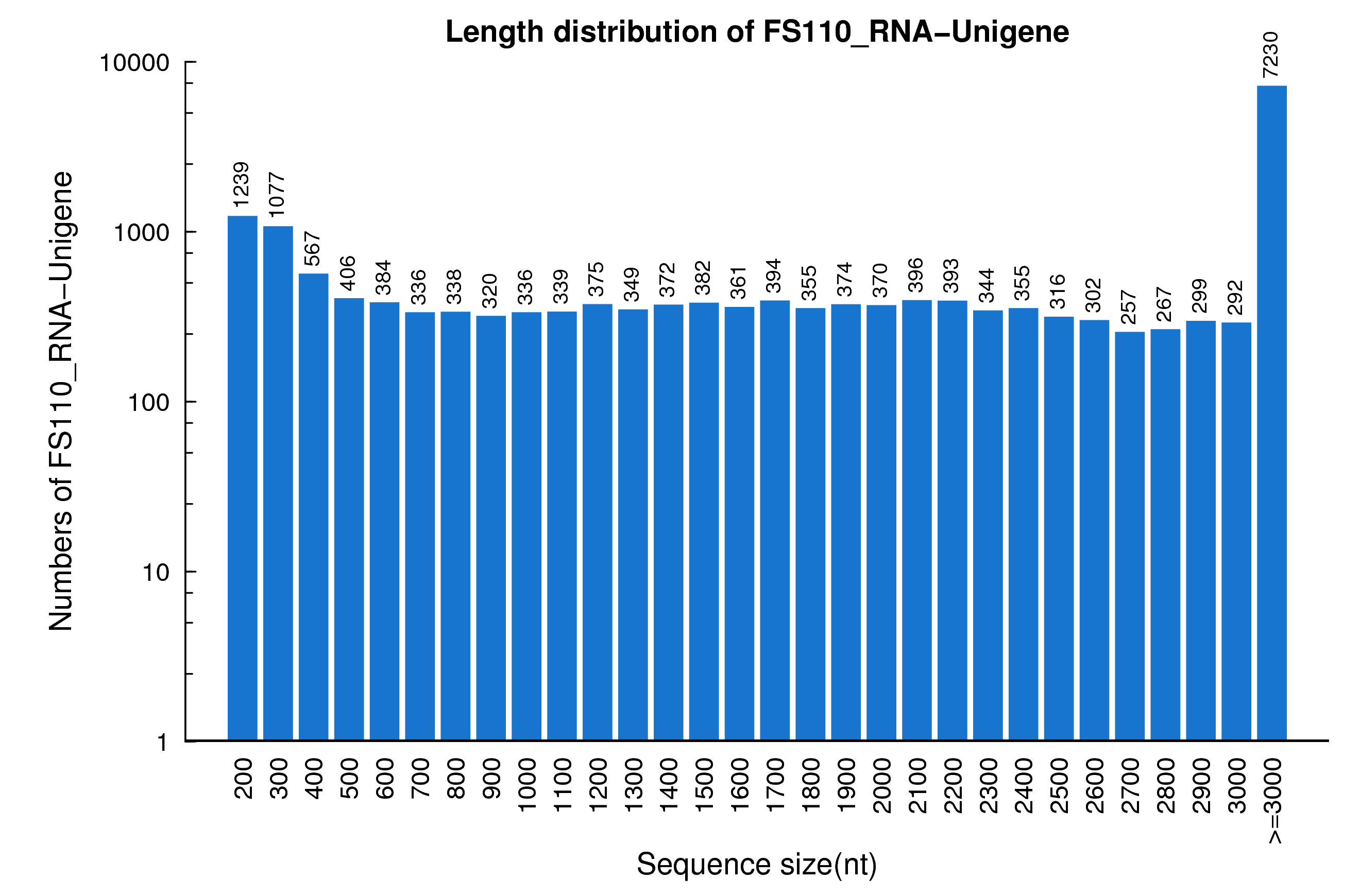

Supplement: Supplementary file 1 [file ijms-19-01910-s001.zip › Figure S1 Lenth distribution unigenes of FS110 transcriptome.tif]

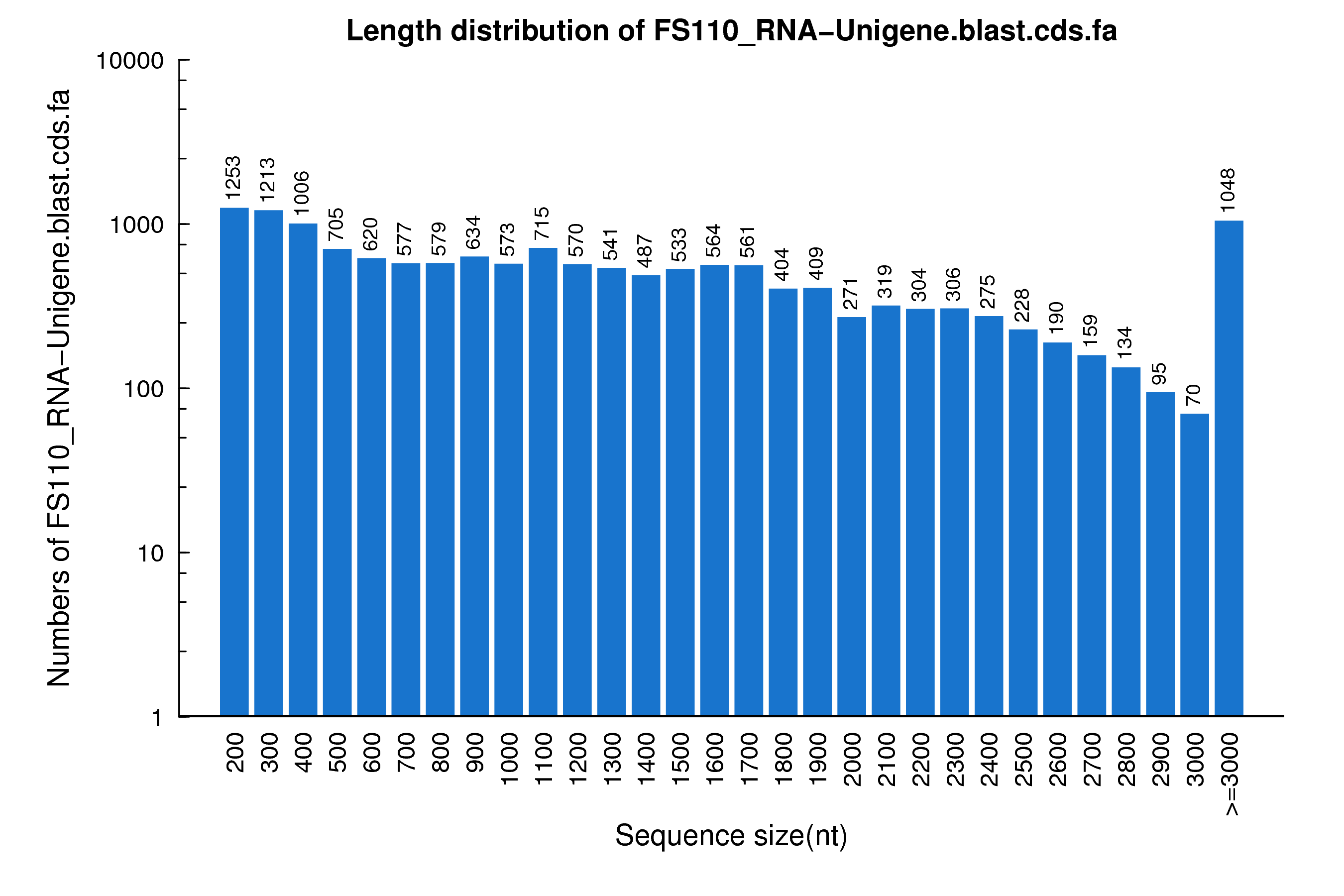

Supplement: Supplementary file 1 [file ijms-19-01910-s001.zip › Figure S2 Lenth distribution of CDS of FS110 transcriptome.tif]

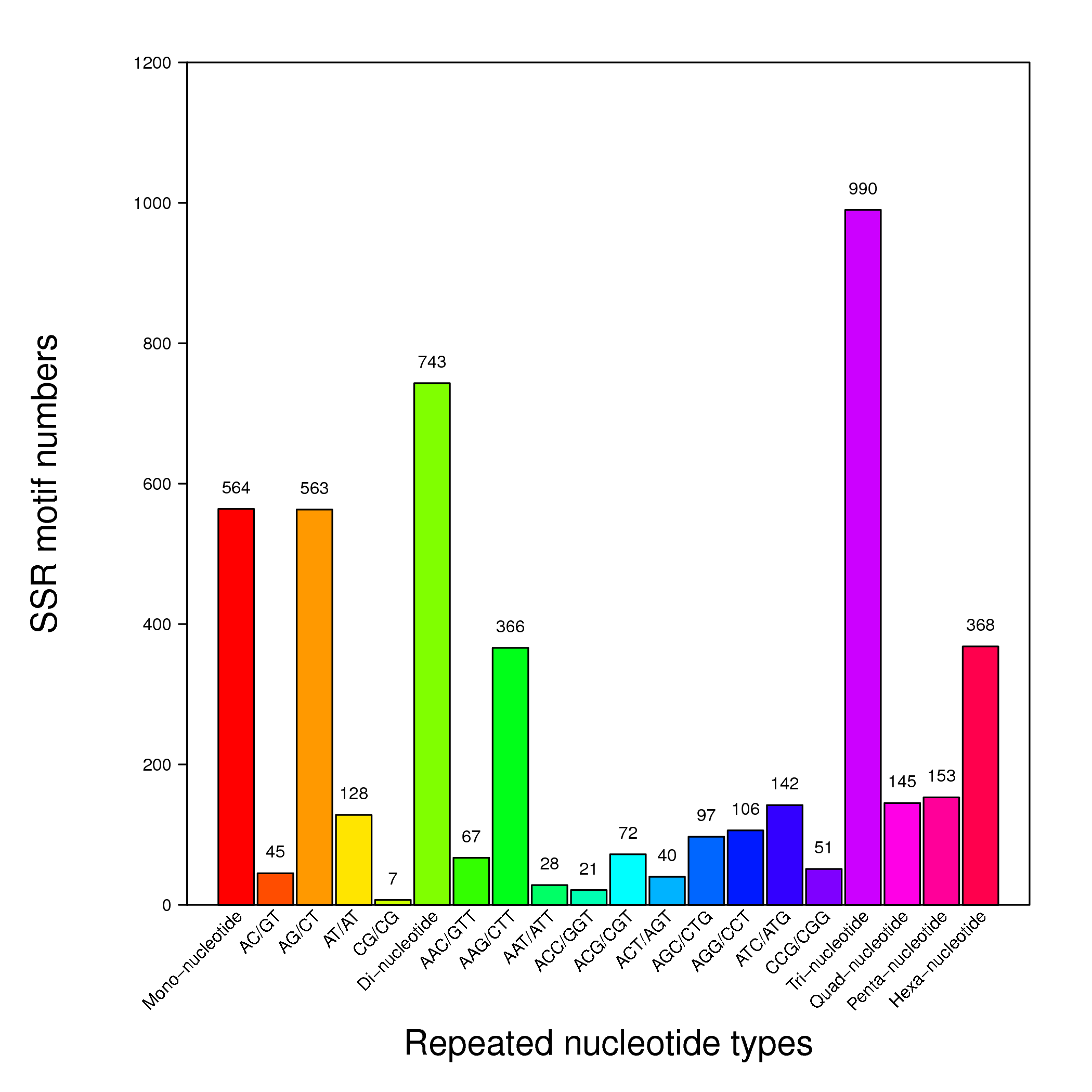

Supplement: Supplementary file 1 [file ijms-19-01910-s001.zip › Figure S3 The SSR distribution of FS110 transcriptome.tif]
